# Pleural thickening induced by *Glaesserella parasuis* infection was linked to increased collagen and elastin

**DOI:** 10.3389/fcimb.2022.952377

**Published:** 2022-08-11

**Authors:** Huimin Gong, Liying Chen, Yanling He, Kexin Hua, Bin Ma, Yuan Gao, Xiaojuan Xu, Xueying Hu, Hui Jin

**Affiliations:** ^1^ State Key Laboratory of Agricultural Microbiology, Huazhong Agricultural University, Wuhan, China; ^2^ College of Animal Medicine, Huazhong Agricultural University, Wuhan, China; ^3^ Hubei Provincial Key Laboratory of Preventive Veterinary Medicine, Huazhong Agricultural University, Wuhan, China; ^4^ Key Laboratory of Preventive Veterinary Medicine in Hubei Province, The Cooperative Innovation Center for Sustainable Pig Production, Wuhan, China

**Keywords:** *Glaesserella parasuis*, Glässer’s disease, pleural thickening, collagen, elastin

## Abstract

*Glaesserella parasuis* is well-known for causing Glässer’s disease, which costs the worldwide swine industry millions of dollars each year. It has been reported the symptom of pleural thickening during Glässer’s disease but this symptom has received little attention. And there is no research on the elements which promote pleural thickening. In this study, pleural thickening was discovered to be associated with increased collagen fibers and elastic fibers. Furthermore, collagen-I and elastin were found to be up-regulated and concentrated in the pleura at the mRNA and protein levels following infection. To summarize, our findings add to the theoretical understanding of Glässer’s disease and provide strong support for further research into the pathogenic mechanism of *Glaesserella parasuis* and the program’s target treatment.

## Introduction

The NAD-dependent gram-negative bacterium *Glaesserella parasuis* (*G. parasuis*) belongs to the family *Pasteurella* (Nedbalcova et al., 2006; Ni et al., 2020). Its infection causes Glässer’s disease, which has a high incidence and mortality, making a substantial impact on the healthy development of the worldwide pig industry with massive economic losses (Nedbalcova et al., 2006; Huang et al., 2020). Glässer’s disease primarily attacks nursing piglets and clinical symptoms of affected pigs include fever, dyspnea, cough, joint swelling, and neurological symptoms (Pereira et al., 2017; Dazzi et al., 2020). The most common significant features of Glässer’s disease are polyserositis, pericarditis, arthritis, and meningitis (Nedbalcova et al., 2006; Brockmeier et al., 2013). Polyserositis caused by *G. parasuis* infection is generally accompanied by the presence of fibrinous exudate and even pseudomembranes on the surface of thoracic and abdominal organs which significantly affect the normal physiological function of the respiratory system (Vahle et al., 1995; Ramirez, 2018). However, there is currently no effective drug for the targeted treatment of polyserositis.

Polyserositis can lead to fibrinous exudation which is generally thought to be the consequence of the deposition of fibrin and the accumulation of the immune cells on the surface of the organ (Vahle et al., 1995). In addition to fibrinous exudation, pleural thickening has been observed in polyserositis during Glässer’s disease (Bom et al., 2020). Visceral pleura contains a large number of collagen and elastin (Lu et al., 2022). Collagen (COL) is the most abundant protein among mammals with a characteristic canonical right-handed triple-helical domain consisting of three polypeptides (Ricard-Blum, 2011; Vaca et al., 2020). The collagen family has been identified with 28 members currently, which is essential for cell growth and differentiation, tissue structure, and wound healing (Ricard-Blum, 2011; Mathew-Steiner et al., 2021). Collagen has also been proved to be one of the anchored targets of specific host macromolecules for pathogen colonization with the most common types observed being collagen I, II, III, IV, and V (Vaca et al., 2020; Arora et al., 2021). Furthermore, the way bacteria adhere to collagen has been extensively researched (Zong et al., 2005; Ross et al., 2012; Herman-Bausier et al., 2016; Arora et al., 2021). Bacteria bind to collagen primarily *via* specific membrane proteins such as microbial surface components recognizing adhesive matrix molecules (MSCRAMMs) and M-like protein (Arora et al., 2021).

Elastin (ELN) is a crucial component comprising elastic fiber and it is formed through cross-linking of small soluble precursor tropoelastin encoding by ELN with the modification of lysyl oxidase (LOX) (Wang et al., 2021). Mature elastin is insoluble and long-lived with slowly degradation rate and low expression level of ELN in normal conditions (Wang et al., 2021). It is widely distributed and contributes to tissue elasticity and resilience (Uitto et al., 2013). Similar to collagen, elastin also can serve as the interaction object mediating bacteria colonization. It has been proved that *Mycobacterium tuberculosis* and *Staphylococcus aureus* make use of elastin for colonization and invasion (Downer et al., 2002; Kuo et al., 2013).

Even though it has been reported the phenomenon of pleural thickening during Glässer’s disease (Bom et al., 2020). The phenomenon has received little attention. Here we adopted 25-day-old piglets to establish the 1-day infection model of *G. parasuis* with high-virulent strain SH0165 and discovered pleural thickening on the lung. On the one hand, increased collagen fiber and elastic fiber were shown to be correlated with pleural thickening according to Masson’s Trichrome Staining and Modified Gomori’s aldehyde-fuchsin staining. It was further demonstrated by RT-qPCR and immunofluorescence that pleural thickening was related to collagen-I and elastin. On the other hand, fibrin(ogen) was found concentrated on the pleura and increased in mRNA and protein expression levels after infection. Our results lay a theoretical foundation for further elucidating the molecular pathogenesis of *G parasuis* and for targeted therapy Glässer’s disease provides important direction.

## Materials and methods

### Animal experiment

Six 25-day-old piglets were randomly separated into two equal groups. One group was infected with *G. parasuis* strain SH0165, a highly virulent strain of serovar 5, which was resuspended in 2 mL phosphate-buffered saline (PBS) at the dose of 2×10^9^ CFU through intraperitoneal injection, whereas the control group was injected with comparable PBS. One day after infection, when the infection group piglets developed significant clinical symptoms of Glässer’s disease including high fever, limp, trouble breathing, all six piglets were killed with pentobarbital. Lung tissue from each piglet was swiftly taken and divided into two parts; one was immediately snap-frozen in liquid nitrogen and kept at -80°C for long-term storage, while the other was fixed in 4% formaldehyde for paraffin embedding. For colony counting, approximately 0.2g of each piglet’s lung tissue was collected and homogenized. After that, tissue homogenates were applied for serial dilution and plated on the TSA plates (containing 5% newborn bovine serum and 1% Nicotinamide adenine dinucleotide). After an overnight incubation at 37°C, the number of colonies was counted to determine the bacterial burden enumeration. Three duplicates were carried out in each piglet.

### Immunohistochemistry

Immunohistochemistry was performed to detect the accumulation of *G. parasuis*. In brief, after antigen retrieval, sections were exposed in 3% hydrogen peroxide for peroxidase quenching. The slides were then blocked with 1% bovine serum albumin and treated overnight at 4°C with porcine polyclonal anti-*G. parasuis* type V antibody (1:50, courtesy of PhD Xiaojuan Xu). HRP goat anti-swine IgG (ANT017, Antgene) was served as secondary antibodies for detection. Images were captured using Nikon (Tokyo, Japan) Eclipse Upright microscope.

### Histology and immunofluorescence

For general histology, in paraffin sections from lung tissue samples, Hematoxylin and eosin (H&E) staining were performed according to standard steps.

For immunofluorescence, 6-μm sections were treated with xylene and graded alcohols. Antigen retrieval was accomplished by microwaving slides in EDTA antigen retrieval buffer (pH 8.0). The slides were blocked with 5% BSA for 1 **h** and then incubated overnight at 4°C with rabbit polyclonal anti-collagen-I antibody (1:50; NB600-408; NOVUS), mouse monoclonal anti-elastin antibody (1:50; NB100-2076; NOVUS), or mouse anti-Fibrinogen beta chain (FGB) antibody (1:50; M01204-1; Boster), followed by Alexa Fluor 488-conjugated goat anti-rabbit secondary antibodies (A-11008, Invitrogen) or Alexa Fluor 488-conjugated goat anti-mouse secondary antibodies (A32723, Invitrogen) for 1h at room temperature and nuclei were stained with DAPI (62248, Invitrogen). The Pannoramic 250 Flash III (3DHISTECH) was used to scan the sections, and CaseViewer 2.4 was used to record the images.

### Masson’s trichrome staining

To determine whether or not collagen fiber deposition occurs during *G. parasuis* infection, Masson’s Trichrome Staining was carried out exactly as described previously (Kraya et al., 2019). In brief, the paraffin slices were stained at room temperature with Weigert’s Iron Hematoxylin, Beibrich Scarlet-Acid Fuchsin, Phosphotungstic acid, and Aniline Blue before being rinsed with Acetic Acid Working solution and dehydrated. The images were captured with Olympus BX53 microscope (Olympus).

### Modified Gomori’s aldehyde-fuchsin staining

Gomori’s aldehyde-fuchsin staining was used to detect elastic fiber according to the manufacturer’s instructions (G1593, Solarbio). In a nutshell, the 7-μm slices were cut from paraffin-embedded lung tissues using the microtome. After being deparaffinized and rehydrated, the slides were treated with the Acid Oxidizing Solution for 5 minutes and the Acid Bleach Solution for 5 minutes before being rinsed with 70% alcohol. The slices were then stained for 10 minutes with the aldehyde-fuchsin solution and then with orange G solution before being dehydrated and transparent.

### RNA extraction and RT-qPCR

The total RNA of each lung tissue sample was isolated using TRIzol (Invitrogen). The purified RNA was quantified *via* Nanodrop 2000 (Thermo Scientific) and 1 μg RNA from each sample was reverse transcribed to cDNA using the reverse transcription kit (Takara). The RT-qPCR was conducted using the SYBR Green Master Mix according to the manufacturer’s instructions (Takara). The primer sequences used were presented in **Table 1** and the expression levels of the detected gene were normalized to the expression level of GAPDH according to the 2^−ΔΔCt^ method. The data was recorded as means ± SD, and statistical analyses were performed using the GraphPad Prism 6 software. P values were calculated using Student’s t-tests to assess significant differences (ns: not significant, *p < 0.05, ***p < 0.001).

**Table 1:** qPCR primers used in this study.

Gene	Accession Number	Primer Sequence (5’-3’)	Amplified length
FGA	XM_021101484	F: CTCCTGCAGATTTCAAGAC R: CCTGGTCCATGAGATATAGA	142bp
FGB	NM_001244113	F: ACAGGATGTCAGTTGCAAGA R: AGGACTGGGAAACAGAGTCT	100bp
FGG	NM_001244524	F: GGCAGTGAGGATTTCAAG R: CAGACTGTGTGGTTATCAGA	124bp
COL-I	XM_021067155	F: CCTGGACGCCATCAAAGTCT R: CTTGCTGATGTACCAGTTCTTCTG	100bp
COL-II	XM_021092611	F: GCAAGGACAAGAAACACATC R: TGGACGTTGGCAGTGTTAG	100bp
COL-III	NM_001243297	F: GAGGATGCTCCCATCTTGGT R: TCGCAGAGAACAGATCCTGAGT	100bp
COL-V	XM_021074694	F: GGCACTATGATGAGAGCAT R: GACTTCAGAGTAGCATGGA	109bp
COL-XXIV	XM_021090341	F: GTTACAGTCCCGTAAAGTC R: CACCACTAACCTCTTAGGTA	100bp
COL-XXVII	XM_021067868	F: GTGGACAGACGTGTCTCAA R: GTCACCTCAGAGCTTAGCA	100bp
ELN	XM_021085777	F: AACTCGGAGCAGGAGTTGGA R: CTTGACTCCTGTTCCAGTGG	155bp
GAPDH	NM_001206359	F: CCCCAACGTGTCGGTTGT R: CCTGCTTCACCACCTTCTTGA	83bp

## Results

### *G. parasuis* infection inducing pleural thickening

**Figure 1:**
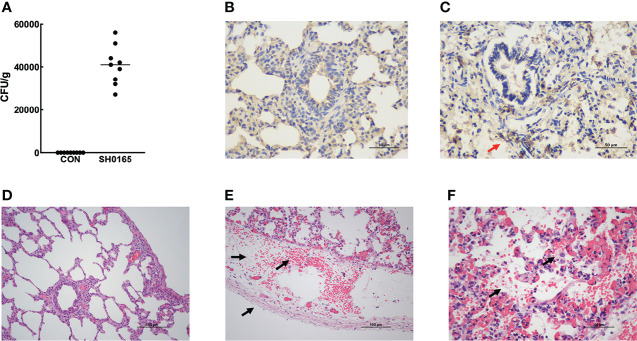
Establishment of G parasuis infection model and Examination of pathological changes in lung induced by *G parasuis* high-virulence strain SH0165. **(A)** Colony counts of *G parasuis* in lung tissue. **(B, C)** Immunohistochemistry to detect *G parasuis* in lung tissue. **(B)** Control group. **(C)** SH0165 infection group. The accumulation of bacteria was indicated by red arrows. After infection with SH0165 at 24h post-infection, lung tissues were collected for H&E staining. Piglets were injected with PBS as a control. Compared with the control group **(D)**, various pathological changes were observed in the section of the *G parasuis* infection group: **(E)** bleeding, pleural thickening, lymphatic dilatation (black arrows), **(F)** fibrinous exudation in the alveolar space, macrophage infiltration which were indicated by black arrows. Scale bar: 50μm **(B, C, F)** and 100μm **(D, E)**.

After euthanizing piglets, colony counting and IHC were performed to detect the existence of *G. parasuis.* The results of colony counts showed that the bacterial load in pig lungs was about 4×10^4^ CFU/g after infection (**Figure 1A**). And the results of immunohistochemistry further demonstrated the accumulation of *G. parasuis* (**Figures 1B, C**). The above results prove the establishment of the *G. parasuis* infection model. Following that, the lungs were fixed for H&E staining. As shown in **Figures 1D–F**, pleural thickening with bleeding and lymphangiectasia was observed in the *G. parasuis* infection group compared to the control group (**Figures 1D, E**). And some alveoli were invaded with fibrinous exudates and inflammatory cells (**Figure 1F**).

### Pleural thickening induced by *G. parasuis* was related to the deposition of collagen fiber and elastic fiber

**Figure 2:**
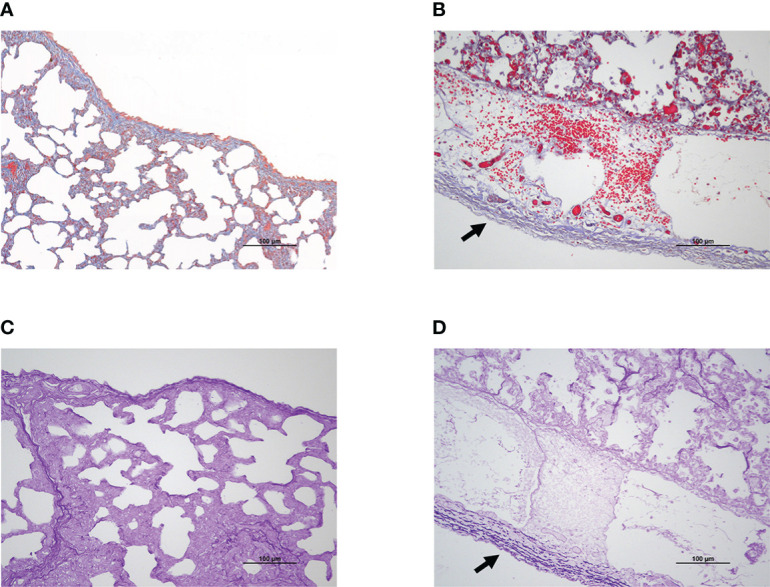
Pleural thickening was related to collagen fiber and elastic fiber deposition during SH0165 infection. **(A, B)** Collagen fiber increased and enriched on pleura after SH0165 infection. Collagen fiber was indicated as blue through Masson’s Trichrome staining. **(A)** Control group. **(B)** SH0165 infection group. The increase of collagen fiber following SH0165 infection was highlighted by black arrow in **(B)**. **(C, D)** Elastic fiber elevated and accumulated on pleura after SH0165 infection. The elastic fiber was shown as deep purple according to Modified Gomori’s aldehyde-fuchsin staining. **(C)** Control group. **(D)** SH0165 infection group. The enrichment of elastic fiber after SH0165 infection was indicated with black arrow in **(D)**. Scale bar: 100μm.

Since the pleura possesses a large number of collagen and elastic fibers, Masson’s trichrome staining and modified Gomori’s aldehyde-fuchsin staining were performed to determine if *G. parasuis* infection impacted the content and distribution of these two fibers in lung tissue. Collagen fiber exhibited blue deposition in Masson trichrome staining (**Figures 2A, B**), while elastic fiber showed deep purple fiber in modified Gomori’s aldehyde-fuchsin staining (**Figures 2C, D**). According to the results, *G. parasuis* infection enhanced the content of collagen fiber and elastic fiber on serosa (**Figure 2**). This suggests that collagen fibers and elastic fibers play an important role in pleural thickening induced by *G. parasuis* infection.

### Fibrin(ogen), collagen and elastin were up-regulated in mRNA levels after *G. parasuis* infection

**Figure 3:**
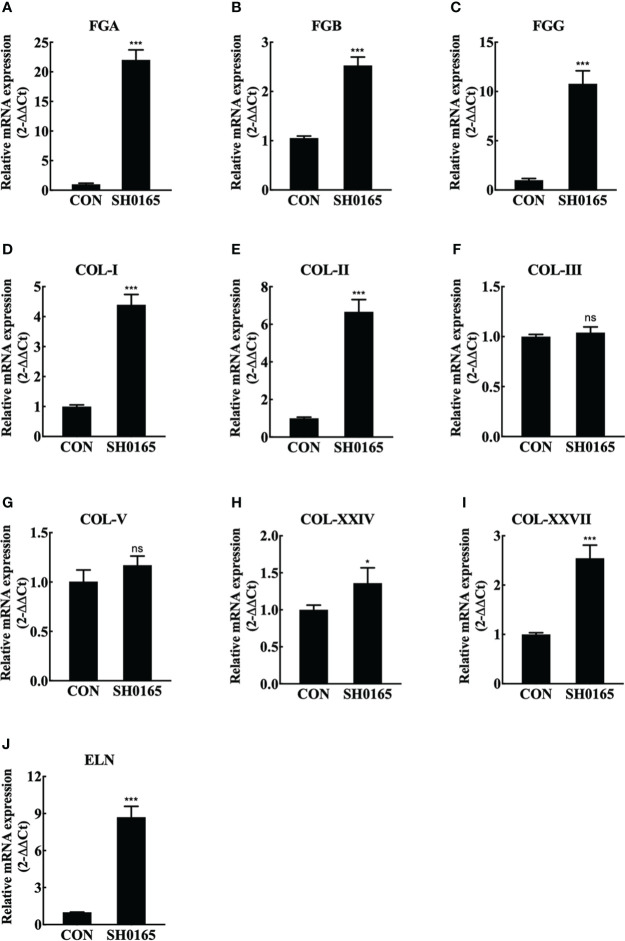
SH0165 infection up-regulated fibrin(ogen), fibril-forming collagen and elastin in mRNA expression level. **(A-C)** fibrin(ogen) gene. **(D-I)** Fibril-forming collagen subfamily member. **(J)** ELN. Data were expressed as mean ± SD (ns: not significant, *p < 0.05, ***p < 0.001).

Lungs were sampled for RNA extraction and real-time RT-PCR to further compare the mRNA expression levels of elastin and collagen before and after *G. parasuis* infection. Considering that fibrin(ogen) was the main ingredient of fibrinous exudation induced by *G. parasuis* infection, the fibrin(ogen) genes were also detected. As shown in **Figures 3A–C**, fibrinogen alpha chain (FGA), FGB, and fibrinogen gamma chain (FGG) all were up-regulated by roughly 22 times, 2.6 times, and 11 times respectively. The observation that all of the peptide chains that comprise fibrin(ogen) were up-regulated suggested that more raw materials for fibrinogen synthesis could be activated. The collagen family contains 28 members, and the fibril-forming collagens COL-I, II, III, V, XXIV, and XXVII were chosen to detect. The results revealed that the mRNA expression levels of COL-1, COL-II, COL-XXIV, and COL-XXVII elevated by approximately 4 times, 7 times, 1.2 times, and 2.5 times, with respect to the control group. COL-III and COL-V, the other two members, showed no significant changes before and after *G. parasuis* infection (**Figures 2D–I**). ELN, which encodes elastin, the key element of elastic fibers was also detected. In comparison to control, ELN was up-regulated by around 8.5 times in the *G. parasuis* infection group (**Figure 2J**). The ELN and most fibrillar collagen mRNA were up-regulated, suggesting the increase in elastic and collagen fiber synthesis.

### Fibrin(ogen), collagen and elastin were increased in protein level and they all deposited on the serosa after *G. parasuis* infection

**Figure 4:**
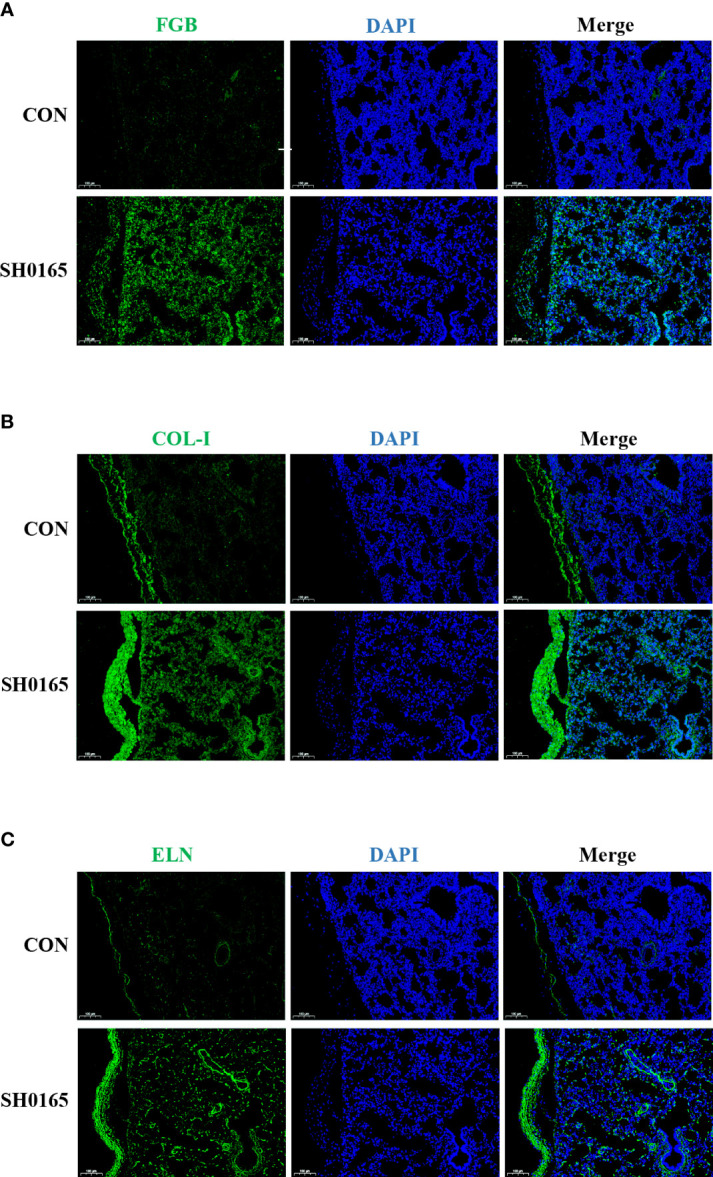
SH0165 infection caused the fibrin(ogen), COL-I and elastin enriched on thickened pleura. The distribution of FGB, COL-I and ELN. The distribution of FGB **(A)**, COL-1 **(B)**, and ELN **(C)** in lung tissue of piglets were detected *via* Immunofluorescence before and after SH0165 infection. Slides of the lung were stained with antibodies of FGB **(A)**, COL-1 **(B)** or ELN **(C)** (green), and DAPI (blue). Scale bar: 100μm.

To compare the protein expression level and spatial distribution of fibrin(ogen), collagen, and elastin before and after *G. parasuis* infection, lung tissue samples were used to perform immunofluorescence staining. FGB was utilized to demonstrate the distribution of fibrin(ogen). There was almost no green fluorescence observed in the control. After infection, the expression of FGB was increased on the lung and both sides of the serosa (**Figure 4A**). Because COL-I was the most abundant member of the fibril-forming collagen subfamily, it was chosen to represent the distribution of fibril-forming collagen. COL-I was predominantly observed in the serosal membrane in the control lung tissue. In the *G. parasuis* infection group, COL-I expression was considerably higher in the lung and enriched in the thickened serosal membrane compared to the control group (**Figure 4B**). In terms of elastin staining, the control possesses a low expression level of elastin, which is mostly located as a thin layer on the serosal membrane on the lung surface. After infection, the proportion of elastin in the lungs increased substantially, and it was more abundant in the serosa (**Figure 4C**). The above findings revealed that *G. parasuis* infection could up-regulate the expression and influence the spatial distribution of fibrin(ogen), collagen, and elastin in the lung, which were shown to be highly enriched on the serosa membrane. Fibrin(ogen) was abundant in the pleura according to immunofluorescence staining but no fibrin was observed on the pleura in H&E staining results (**Figure 1B**), indicating that the host was in the early stages of fibrin deposition. Besides, here we first discovered that elevated collagen and elastin levels caused by *G. parasuis* infection accumulated on the serosa membrane, suggesting that they are implicated in pleural thickening in Glässer’s disease.

## Discussion

Glässer’s disease is characterized by fibrinous polyserositis, which results in visible white or yellow fibrinous exudate on the serosal surface (Pereira et al., 2017). In the study, we established the 1-day infection model of Glässer’s disease in 25-day-old piglets through experimental infection of pigs with the high-virulence strain SH0165. According to the H&E staining, only lymphatic dilatation and pleural thickening were observed in the pleura and fibrinous exudate was apparent in some alveolar space (**Figure 1**). RT-PCR and immunofluorescence staining demonstrated that fibrin(ogen) was up-regulated after infection and was molecularly concentrated in the pleura (**Figures 3A–C**, **4A**), suggesting that the host was experiencing the process in which fibrin is just beginning to exude.

However, the causes of pleural thickening in *G. parasuis* infection have yet to be determined. The pulmonary visceral pleura’s natural physiological structure is comprised of extensive collagenous fiber and elastic fiber (Lu et al., 2022), the distribution of which was demonstrated in the current study *via* Masson’s trichrome staining and modified Gomori’s aldehyde-fuchsin staining (**Figure 2**). The finding showed that after infection, both collagen and elastic fibers increased and gathered on the pleura, suggesting that collagen and elastic fibers are indeed linked to pleural thickening. Previous studies reported that fibril forming collagen was highly correlated with a variety of diseases, including skin blistering disease bullous pemphigoid, osteogenesis imperfecta, and Ehlers-Danlos syndrome (Ricard-Blum, 2011). Elastic fiber has been associated with cardiovascular diseases, such as myocardial ischemia-reperfusion and atherosclerosis (Wang et al., 2021). In terms of disease related to both collagenous and elastic fiber, it has been found that collagenous and elastic fiber levels were substantially elevated during interstitial pneumonia, contributing to vascular ECM remodeling and scar formation (Parra et al., 2007). Idiopathic pleuroparenchymal fibroelastosis was accompanied by collagenous and elastic fiber hyperplasia in the visceral pleura, which was primarily in a whirlpool (Cao et al., 2021). Here we first reported that pathogen caused collagen and elastic fibers to be up-regulated and considerably enriched in the pleura, providing new ideas and theoretical foundations for the research of collagen and elastic fiber regulation mechanisms.

Fibril forming collagen subfamily contains collagen I, II, III, V, XI, XXIV, XXVII and collagen I is the most prevalent collagen in the body (Ricard-Blum, 2011). Elastin was the core component of elastic fibers (Wang et al., 2021). The expression of associated genes was also investigated at the mRNA and protein levels. Most fibril forming collagen and elastin were highly up-regulated after *G. parasuis* infection, which was consistent with histology findings (**Figures 3D–J**, **4B, C**). Pathogens have been demonstrated to interact with collagen or elastin for colonization and invasion. Collagen was the binding target for numerous bacterial surface adhesins and virulence factors, such as CNA-Like MSCRAMMs and M-Like proteins, to establish infections in the host (Arora et al., 2021). As for elastin, it has been proposed that *S. aureus* binds to it *via Ebps* for colonization (Downer et al., 2002). Mycobacterial Ag85 proteins interacted with elastin and tropoelastin, indicating that elastin is a crucial ligand in mycobacterial invasion (Kuo et al., 2013).

Here we discovered that *G. parasuis* infection enhanced collagen and elastin levels, which may promote *G. parasuis* colonization and invasion, suggesting new pathways for research into *G. parasuis* pathogenesis and medication development.

## Data availability statement

The original contributions presented in the study are included in the article/supplementary material. Further inquiries can be directed to the corresponding author.

## Ethics statement

The animal study was reviewed and approved by Huazhong Agricultural University’s Scientific Ethics Committee (HZAUSW-2022-0010).

## Author contributions

HJ and HG designed the research. HJ, LC, KH, and YG conducted the experiments. HG and YH acquired the data. HJ, HG, LC, and BM analyzed the data. XX provided porcine polyclonal anti-G. parasuis type V antibody. XH provided guidance on pathology slide preparation. HJ, HG, and KH wrote the manuscript. HJ, HG, XX, and XH revised the manuscript. All authors contributed to the article and approved the submitted version.

## Funding

This work was supported by the National Natural Science Foundation of China (31972643, 31772705), Natural Science Foundation of Hubei Province (2021CFA016), Natural Science Foundation of Hubei Province for Distinguished Young Scholars (2020CFA060), and Applied Basic Research Project of Wuhan (Grant No. 2020020601012254).

## Conflict of interest

The authors declare that the research was conducted in the absence of any commercial or financial relationships that could be construed as a potential conflict of interest.

## Publisher’s note

All claims expressed in this article are solely those of the authors and do not necessarily represent those of their affiliated organizations, or those of the publisher, the editors and the reviewers. Any product that may be evaluated in this article, or claim that may be made by its manufacturer, is not guaranteed or endorsed by the publisher.
